# Directions for Constructing a Cheap Bed and Elastic Frame, for the Easy Conveyance of Sick or Wounded Persons. Invented, and Most Humbly Presented to His Royal Highness the Commander in Chief

**Published:** 1808-08

**Authors:** Patrick Crichton

**Affiliations:** Lieutenant-Colonel of the Second Regiment Royal Edinburgh Volunteers


*Directions for constructing a cheap Bed and elastic Frame, for the easy Conveyance of sick or wounded Persons. Invented, and most humbly presented to his Royal Highness the Commander in Chief, by* PATRICK CRICHTON, *Lieutenant-Colonel of the Second Regiment Royal Edinburgh Volunteers.*

### *Construction of the Elastic Frame.*

THE lower frame is made of ash or elm, seven feet long, and five feet four inches broad.

There are two strong wooden pillars, bound on the sides by two circular pieces of iron, for supporting the elastic frame.

The elastic frame is made of the best ash, supported by the wooden pillars, and semicircular pieces of iron.

The frame or cott, contains a mattrass or pelisse, stuffed with straw.—Two or three hammocks may be suspended, and will answer as well as the cott.

The cott, bed, and mattrass are supported by rings and iron hooks.

Four handles project from the under frame, one foot three inches long each, by which the whole may be carried by four men.

Four semicircular hoops may be placed over the frame, over which a cover can be thrown, to protect the patient from the weather.

The under frame and pillars should be made of ash or elm, well seasoned.

The elastic, or upper frame, should be made of ash, remarkably clean and well seasoned, thick in the middle where it is supported, and tapering towards the ends.

The total expense of the whole, including the iron-work, should not exceed four pounds ten shillings.

### *Directions for using the Bed and Frame.*

The lower frame may be fastened by ropes to any cart or waggon, of the same size, or larger than itself.

The sick or wounded person should first be placed in the bed.

The frame should then be placed over the bed, and the ropes at the head of the bed suspended upon the iron hooks.

Then the ropes at the feet should be hooked up.

The frame, containing one or two sick men, can be easily lifted by the four handles by four men, and carried to any distance to a cart or baggage-waggon.

The

The lower frame is then fixed to the cart by ropes, and the machine is ready to move.

When the sick are taken from the baggage-cart, the whole frame should be lifted at once, and carried to the hospital.

The bed should then be unhooked, first at the feet and then at the head, and the frame taken away.

Upon large English waggons, two or three of these frames may be conveniently placed.

If the carts of any district are too small for the breadth of the frame, it may be made narrower, so as to adapt it to that conveyance.

---

When the machine, was first invented, it was solely intended for the use of the army.

To this purpose it has been successfully applied; and is in common application in several of the garrisons of Great Britain, as affording the easiest means of transporting sick or wounded soldiers, from garrison or quarters, to the hospital.

Since the time of its being adopted by the army, it has likewise been brought into the service of a great many of the public hospitals, not only for the purpose of conveying maimed or bedridden patients from their houses to the wards, but for removing such patients, as were under the necessity of undergoing operations, from the wards to the operation room, and returning them again from the operation room to the wards, without subjecting them to the necessity of being dressed, or even removed from the beds.

Having successfully answered these purposes, it has of late been used, when fixed upon a cart, waggon, or upon the carriage of a postchaise, for removing wounded persons, or such as were afflicted with disease, and who were unable to support the motion of a chaise or coach, from different parts of the country to towns where they might enjoy the benefit of medical advice.

In this manner, the use of it, in Scotland, has become of late very general, and, fortunately, very beneficial to those who have travelled in it; all of them concurring, that they were insensible of any unpleasant motion during their respective journeys.

To enumerate the instances of its successful application in this manner would fill a small volume; but a few facts will enable the public to appreciate its value.

A person was brought in it, with a compound fracture in the the thigh bone, from the west Highlands to Edinburgh, a distance of 74 miles, in two days.

A gentleman, with an attack of gout both in his hands and feet, was removed from Edinburgh to the north of England, above 140 miles, in three days.

In both these instances, and a great many more, the bed and frame were suspended to the carriage of a postchaise, and, with a servant sitting in front, travelled post.

Some hundreds of examples can be adduced of the removal of patients by its means, when fixed on a cart or waggon, and in many of these the patients were in a state of the most severe bodily distress and debility.

In all these removals, the patients have borne testimony to their enduring no additional pain or inconvenience from the motion of the machine; all of them, even in the most severe cases, declaring, that they were alike insensible of bodily fatigue, or of the least increase of pain, from the mode of conveyance.

The royal colleges of physicians and surgeons of this place have bestowed upon it the most unlimited approbation, both by letters addressed to the inventor, and in the publications of several of their members.

In consequence of these proofs of its successful effects, and these encomiums from the learned and respectable bodies who are so well enabled to decide regarding its merits, a number of applications have of late been addressed to the inventor, soliciting him to describe and delineate the machine, so as it might be introduced into general use in the various quarters of the kingdom.

To save time in complying with these requests, he adopted the method of printing and circulating a plate, accompanied with a description, which will clearly demonstrate, at how very small an expence, and with how very little mechanical art, the elastic frame can be constructed; for, in fact, there is no village in Britain, in which an ordinary smith and carpenter reside, where it may not be easily made.

Under these circumstances, the inventor feels it a duty he owes to his country, and to those suffering bodily distress, to give it all the publicity in his power; with which view, and with the most ardent wishes for its continuing to prove beneficial, in mitigating the distresses of such as may require its aid, this account is submitted to the public.

*Gayfield Place, 17th Sept. 1807.*

